# Tri-Modality therapy with I-125 brachytherapy, external beam radiation therapy, and short- or long-term hormone therapy for high-risk localized prostate cancer (TRIP): study protocol for a phase III, multicenter, randomized, controlled trial

**DOI:** 10.1186/1471-2407-12-110

**Published:** 2012-03-22

**Authors:** Hiroyuki Konaka, Shin Egawa, Shiro Saito, Atsunori Yorozu, Hiroyuki Takahashi, Keiko Miyakoda, Masanori Fukushima, Takushi Dokiya, Hidetoshi Yamanaka, Nelson N Stone, Mikio Namiki

**Affiliations:** 1 Department of Integrative Cancer Therapy and Urology, Kanazawa University Graduate School of Medical Science, Kanazawa, Japan; 2 Department of Urology, Jikei University School of Medicine, Tokyo, Japan; 3 Department of Urology, National Hospital Organization Tokyo Medical Center, Tokyo, Japan; 4 Department of Radiology, National Hospital Organization Tokyo Medical Center, Tokyo, Japan; 5 Department of Pathology, Jikei University School of Medicine, Tokyo, Japan; 6 Translational Research Informatics Center, Foundation for Biomedical Research and Innovation, Kobe, Japan; 7 Department of Radiation Oncology, Saitama Medical College, Irima, Japan; 8 Institutes of Preventive Medicine, Kurosawa Hospital, Takasaki, Japan; 9 Department of Urology and Radiation Oncology, Mount Sinai School of Medicine, New York, NY, USA

**Keywords:** Prostate cancer, Trimodality, Radiation therapy, Brachytherapy, External beam radiation therapy, Hormone therapy, Randomized controlled trial, Biochemical progression-free survival

## Abstract

**Background:**

Patients with high Gleason score, elevated prostate specific antigen (PSA) level, and advanced clinical stage are at increased risk for both local and systemic relapse. Recent data suggests higher radiation doses decrease local recurrence and may ultimately benefit biochemical, metastasis-free and disease-specific survival. No randomized data is available on the benefits of long-term hormonal therapy (HT) in these patients. A prospective study on the efficacy and safety of trimodality treatment consisting of HT, external beam radiation therapy (EBRT), and brachytherapy (BT) for high-risk prostate cancer (PCa) is strongly required.

**Methods/Design:**

This is a phase III, multicenter, randomized controlled trial (RCT) of trimodality with BT, EBRT, and HT for high-risk PCa (TRIP) that will investigate the impact of adjuvant HT following BT using iodine-125 (^125^I-BT) and supplemental EBRT with neoadjuvant and concurrent HT. Prior to the end of September 2012, a total of 340 patients with high-risk PCa will be enrolled and randomized to one of two treatment arms. These patients will be recruited from more than 41 institutions, all of which have broad experience with ^125^I-BT. Pathological slides will be centrally reviewed to confirm patient eligibility. The patients will commonly undergo 6-month HT with combined androgen blockade (CAB) before and during ^125^I-BT and supplemental EBRT. Those randomly assigned to the long-term HT group will subsequently undergo 2 years of adjuvant HT with luteinizing hormone-releasing hormone agonist. All participants will be assessed at baseline and every 3 months for the first 30 months, then every 6 months until 84 months from the beginning of CAB.

The primary endpoint is biochemical progression-free survival. Secondary endpoints are overall survival, clinical progression-free survival, disease-specific survival, salvage therapy non-adaptive interval, and adverse events.

**Discussion:**

To our knowledge, there have been no prospective studies documenting the efficacy and safety of trimodality therapy for high-risk PCa. The present RCT is expected to provide additional insight regarding the potency and limitations of the addition of 2 years of adjuvant HT to this trimodality approach, and to establish an appropriate treatment strategy for high-risk PCa.

**Trial registration:**

UMIN000003992

## Background

The majority of low-risk patients with clinically localized prostate cancer (PCa) have a high likelihood of disease-free survival regardless of the treatment option chosen [1]. In contrast, patients with high-risk PCa with high Gleason score, elevated prostate specific antigen (PSA) level, and advanced clinical stage are at greater risk for treatment failure after initial management by single treatment modalities, such as radical prostatectomy (RP), external beam radiation therapy (EBRT), or brachytherapy (BT) [2,3]. Therefore, it is extremely important to establish the most effective and safe treatment strategy for patients with high-risk PCa, preventing local recurrence and biochemical failure at an early stage during treatment. However, high-risk PCa remains a therapeutic challenge for both urologists and radiation oncologists.

As high-risk patients have locally advanced disease with the possibility of direct extension and/or local micrometastases, various combinations of each monotherapy described above have been developed to augment disease-free survival. Recently, several studies concerning radiation therapy (RT)-based trimodality treatment method with BT, EBRT, and hormonal therapy (HT) were reported [4-6]. According to the American Brachytherapy Society (ABS), BT alone is not recommended for high-risk PCa but can be used as a boost in conjunction with EBRT [7]. Thus, the combination of BT and EBRT in this multimodal treatment approach theoretically delivers an escalated dose to the prostate, including extraprostatic extension (EPE) increasing the probability of eradicating all of the local disease. With respect to HT, neoadjuvant and concurrent androgen deprivation also offers both cytoreduction and synergistic enhancement of RT in high-risk PCa; adjuvant HT may play a role in elimination of occult systemic disease and have multiple synergistic effects to radiation on local control due to induction of apoptosis [8,9].

Some previous studies demonstrated a benefit of HT used in conjunction with EBRT to treat locally advanced prostate cancer [10-12]. However, these studies, which demonstrated an advantage with the addition of HT, were done when the radiation dose may have been inadequate to control all local disease. The question therefore that has never been answered is what benefit, if any will long-term androgen deprivation have when much higher radiation doses are delivered. Although the ABS recommends HT in conjunction with BT for cytoreduction of prostate volume, there are no clear indications for using adjuvant HT in intermediate- to high-risk PCa [7]. Moreover, both neoadjuvant and adjuvant HT may significantly induce adverse events, including fatigue, diminished sexual function, and hot flushes and possible early death [13,14]. Accordingly, investigation of the best optimal duration of HT with maximization of outcome while minimizing toxicity is a logical step in the management of localized high-risk PCa, because the prolonged use of HT may result in an increase in adverse events. It is necessary to determine which patients with high-risk PCa will actually benefit from HT despite some compromises in quality of life (QOL) associated with the adverse event profile of this treatment.

As mentioned above, the optimal RT-based trimodality protocol for use in high-risk PCa remains controversial. However, there have been no prospective studies regarding the efficacy and safety of trimodality treatment with combined HT, EBRT, and BT for high-risk patients. Such prospective clinical trials are necessary. Here, we describe our study protocol for high-risk PCa, which is a phase III, multicenter, randomized controlled trial (RCT) of a trimodality treatment protocol with BT using iodine-125 (^125^I-BT), EBRT, and neo-adjuvant and concurrent HT for 6 months with or without adjuvant HT for 2 years. We also assessed whether short-term HT without adjuvant HT or long-term HT with adjuvant HT is better with regard to improved local control and biochemical cure rate in high-risk localized PCa. The final goal of this study is to establish an appropriate treatment strategy for high-risk PCa without increasing the occurrence of adverse events.

## Methods/Design

### Aim of the study

To evaluate the efficacy and safety of long-term *vs*. short-term HT in the setting of trimodality therapy with ^125^I-BT, EBRT, and HT in high-risk PCa patients.

### Study design

**Figure 1:**
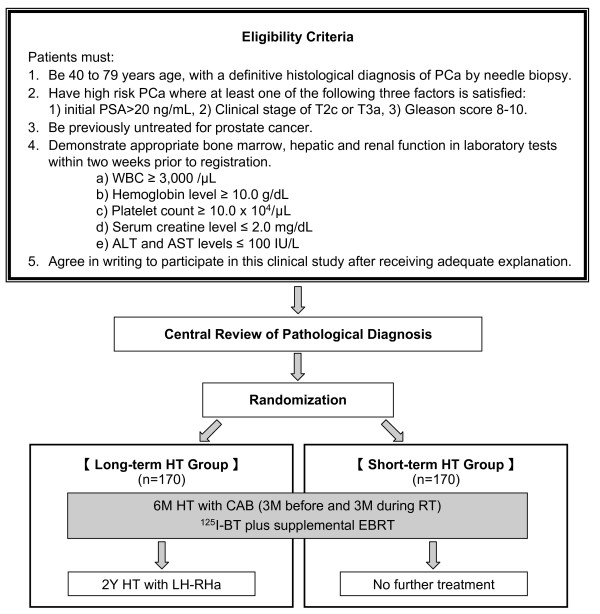
**Study design of TRIP (UMIN000003992)**.

The present study is a phase III, multicenter, RCT of a trimodality treatment protocol with ^125^I-BT plus supplemental EBRT with short- or long-term HT for patients with untreated high-risk PCa. We will randomly assign patients with high-risk PCa who have received ^125^I-BT and supplemental EBRT plus 6 months of HT with combined androgen blockade (CAB) into two groups: one receiving no further treatment and another receiving 2 years of HT with a luteinizing hormone-releasing hormone agonist (LH-RHa) (Figure 1).

### Additional measures

Two validated QOL questionnaires, the SF-8™, which has been translated into Japanese, and the Expanded Prostate Cancer Index Composite (EPIC), will be administered prior to hormonal therapy and at the 60 months after the beginning of CAB to comprehensively cover the various aspects of physical and psychosocial wellbeing.

### Site selection

For an institution to participate, the institution's attending physicians must:

a. Acquire certified documentation of participation in the Japanese Brachytherapy Scientific Meeting's training session for the ^125^I-BT procedures.

b. Have experience with ^125^I-BT in at least 50 patients.

c. Regularly perform ^125^I-BT procedures.

### Eligibility criteria - Inclusion criteria

Patients must:

a. Be from 40 to 79 years of age when obtaining written informed consent.

b. Have high-risk PCa with a definitive histological diagnosis by needle biopsy. In this study, high risk is defined as those cases in which where at least one of the following three conditions is satisfied: 1) PSA > 20 ng/mL prior to CAB, or 2) clinical stage of T2c or T3a, or 3) Gleason score ≥ 8 as determined by central pathological judgment.

c. Performance status of 0-1, according to the Eastern Cooperative Oncology Group.

d. Previously untreated for PCa.

e. Appropriate bone marrow, hepatic, and renal function as demonstrated in laboratory tests within two weeks prior to registration.

a) WBC ≥ 3000/μL.

b) Hemoglobin level ≥ 10.0 g/dL.

c) Platelet count ≥ 10.0 × 10^4^/μL.

d) Serum creatine level ≤ 2.0 mg/dL.

e) ALT and AST levels ≤ 100 IU/L.

f. Computed tomography (CT) and bone scan without evidence of metastases

### Eligibility criteria - Exclusion criteria

Patients are ineligible if they:

a. Have previously received surgery and/or hyperthermia for BPH.

b. Exhibit clinical stage ≥ T3b.

c. Have a second cancer that requires treatment.

d. Have collagen diseases.

e. Have poorly controlled ischemic cardiac disease.

f. Have poorly controlled hypertension (*i.e*., diastolic pressure ≥ 120 mmHg)

g. Have a severe psychiatric disorder, including schizophrenia and dementia.

h. Have poorly controlled diabetes.

i. Are using steroid drugs other than topical ointments.

j. Are using antiandrogenic therapy.

k. Are considered by a principal or clinical investigator to be inappropriate for participation in the present study for any other reason.

### Informed consent - ethics approval

The study was conducted in accordance with the Declaration of Helsinki 1975, as revised in 2000. All treatments for prostate cancer are undertaken following written informed consent, and further consent is obtained for procedures to confirm the high-risk diagnosis. This study received approval from the Foundation for Biomedical Research and Innovation, Translational Research Informatics Center (TRI) ethical review committee (approval No. 10-12, date Sep 10th, 2010) and the institutional ethics committees of the participating institutions.

### Methods of recruitment and random allocation

Recruitment of patients is supported by the tri-modality therapy with ^125^I-BT and EBRT and short- or long-term HT for High-risk Localized PCa (TRIP) Study Group. Recruiting began in October 2010, and is planned for completion by September 2012. All prostatic biopsy histological slides of newly diagnosed prostate cancer since the initiation of the study have been reviewed by central pathologists, with patient eligibility determined at the time of review. Eligible patients are randomly assigned to one of two treatment arms through the data center at the TRI. Randomization is done centrally using a minimization method to obtain good between-group balance for factors including age category (< 70/≥ 70), PSA category (≤ 20/> 20 ng/mL), Gleason score by central pathological judgment category (< 8/≥ 8), and institutions.

### Technique of ^125^I-BT

^125^I-BT for all patients is administered using an ultrasound-guided technique with the Mick applicator [15]. The implant is planned to deliver a prescription dose of 110 Gy or more to the clinical target volume (CTV), which includes the prostate gland and treatment margin [16]. Although individual technical aspects are institution-dependent, efforts are made to ensure optimal quality control of the radiation dose. Standardization included training in real-time computer assisted intraoperative technique [17], three types of I-125 activity levels based on prostate volume (< 15 cc; 0.25-0.29 mCi, 15-40 cc; 0.3-0.34 mCi and > 40 cc; 0.35-0.4 mCi), prostate D90 (the mean dose received by 90% of target volume) > 110 Gy, V100 (the mean volume receiving 100% of the prescription dose) > 95%, V150 (the mean volume receiving 150% of the prescription dose) < 60%, urethral V150 (mL) = 0 mL and maximum urethral dose < 220 Gy, rectal V100 (mL) < 0.1 mL. CT images, taken at 2-5 mm intervals, are obtained within 3-7 weeks after ^125^I-BT to determine dose-volume histogram (DVH) for the prostate, urethra, and rectum [16,18]. We organized a quality control committee for this study to assess the variance of postimplant dosimetry. This board will meet regularly while this protocol is running to monitor and compare dosimetry. Although the prescription dose for the I-125 is 110 Gy, the board recognizes that post-implant D90 may vary. One of the goals of the study is not to have local failure result from too low a dose of delivered dose. Therefore, as long as the rectal V100 < 1.0 mL and the urethral D10 (the mean dose received by 10% of target volume) < 200 Gy each center will be allowed to adjust the EBRT total dose if the I-125 D90 < 100 or > 140 Gy. The results of comparative analysis will be reported separately.

### Supplemental EBRT

From 4 to 8 weeks after ^125^I-BT, 45 Gy of EBRT in 25 fractions is routinely delivered by three-dimensional conformal radiation therapy (3D-CRT) using ≥ 6 MV photons or intensity-modulated radiation therapy (IMRT). Generally, a daily fraction of 1.8 Gy will be administered 5 days per week for 5 weeks. If the D90 form the implant is < 100 Gy up to the total dose of 50.4 Gy (1.8 Gy × 28) and if the dose is > 140 Gy the EBRT will be reduced to 39.6 Gy (1.8 Gy × 22).

3D-CRT will be delivered with 4 or more fields isocentric beam setup based on CT. The technical aspects of IMRT delivery are institution-dependent.

The clinical target volume (CTV) is defined as the prostate and the proximal seminal vesicle (SV) within 1 cm from the prostate-SV junction [19]. No attempt is made to treat the pelvic lymph nodes. A planning target volume (PTV) is applied to the CTV such that there is a block margin of 1.5-2 cm around the CTV. Position verification and correction are performed by standard port film imaging in the case of 3D-CRT and with orthogonal film isocenter verification in the case of IMRT.

### Contents of HTx

HT with CAB consists of a subcutaneous LH-RHa administration in conjunction with oral antiandrogen for 3 months before and continuing for 3 months after ^125^I-BT. After 6 months of HT, the patients were assigned to receive no further HT treatment or to continue HT with the same LH-RHa but without the antiandrogen for an additional 2 years. Bicalutamide (80 mg/day) is used as an oral antiandrogen, and either goserelin acetate (3.6 mg per month or 10.8 mg every 3 months) or leuprolide acetate (3.75 mg per month or 11.25 mg every 3 months) is subcutaneously injected as LH-RHa.

### Data collection

This study design was chosen to ensure accurate, standardized, and high-quality data collection. All patients giving written informed consent to the study are asked to complete a short family history and epidemiology questionnaire. Electronic Data Capture (EDC) systems are used to collect clinical data in electronic format, with clinical data being obtained from patient medical records by the Translational Research Informatics Center. A follow-up data form is completed by the Clinical Trials Practitioner (CTP) at diagnosis, 3, 6, 9, 12, 15, 18, 21, 24, 27, and 30 months, and then every 6 months until 84 months from the date of commencement of CAB. These forms capture information regarding patient characteristics, disease presentation, diagnosis and treatment, PSA, recurrence and survival. Annual follow-up is continued until death, loss to follow-up, or the end of the active phase of the study (September 2022).

### Definition of endpoints

The primary endpoint is biochemical progression-free survival (bPFS). Biochemical progression is defined as an increase in PSA of ≥ 2 ng/mL from the nadir value following treatment. Secondary endpoints are: 1) OS, 2) clinical progression-free survival (local progression, distant failure), 3) disease-specific survival (DSS), 4) salvage therapy non-adaptive interval, 5) QOL, and 6) adverse events. OS and PFS are calculated from the 1st day of treatment to death from any cause and or to identification of disease progression or death, respectively. Local progression is defined as local tumor reappearance at the primary site. Local tumor reappearance will be confirmed by rectal examination and imaging studies, such as magnetic resonance imaging (MRI) or CT or biopsy when indicated. The primary endpoint and the secondary endpoints are the bPFS at 7 years and OS (or DSS) at 10 years, respectively, following initial HT with CAB to investigate the relationship between results and eventual recurrence after completion of trimodality therapy.

### Planned statistical analyses

It has been shown that the 7-year bPFS rate ranges from 67.8% to 83% in patients with high-risk PCa who undergo combination of BT and EBRT or trimodality treatment [5,20]. Based on these two reports, the 7-year bPFS rate of the short-term HT group is estimated to be 75%. On the other hand, the 7-year bPFS rate of the long-term HT group is estimated to be 87.5% (*i.e*., hazard ratio = 0.464) based on three RCTs comparing EBRT plus long-term HT with EBRT alone [10-12]. Taken together, 153 patients for each group are needed to detect a significant difference between treatments by log-rank test with a significance level of 0.05 and a power of 80%. Given the further assumption that approximately 10% of randomized patients will not be evaluable for various reasons, the target sample size was set at 170 patients per group (340 total).

Statistical analyses will be performed on an intention-to-treat basis. Survival curves will be estimated using the Kaplan-Meier method. The log-rank test will be used to test for differences in survival curves between the two groups of patients. The hazard ratio will be estimated using the Cox proportional hazard model. The longitudinal change of QOL scores (IPSS, SF-8™, EPIC) between diagnosis and 60 months following ^125^I-BT will also be compared between groups. Patients will be evaluated for toxicity, graded according to the National Cancer Institute Common Toxicity Criteria version 4.0 (http://evs.nci.nih.gov/ftp1/CTCAE/About.html). For all patients, the incident proportion of grade 3 adverse events will be compared between groups by Fisher's exact test. All tests will be two-sided, and a *P*-value of 0.05 will be considered statistically significant. Five years after the last patient is recruited, an interim analysis will be performed and the results will be reported to the Independent Data Monitoring Committee.

### Patient enrollment and anticipated completion of enrollment

Our current expectation is that the final patient will be enrolled by September 2012, the study will be clinically complete by 2022, and the results will be available during the first quarter of 2023. Monthly enrollment was going well for the goal of a total of 340 cases, actually, cumulative enrollment reached 130 cases in September 2011.

## Discussion

High-risk PCa features are associated with poor pathological outcomes after RP [3,21]. Increasing Gleason score, high PSA level, and advanced clinical stage have all been shown to be correlated with EPE, SV invasion, and positive surgical margins. Both Partin's tables and Naito's Japanese nomograms have demonstrated the increased incidence of the above-mentioned pathological findings with higher risk features [22,23]. These pathological findings have also been shown to be associated with higher rates of biochemical failure [21,24]. Explanations for these outcomes have focused on the presence of microscopic dissemination of cancer cells at initial diagnosis. For this reason, little attention has been focused on optimizing local control and more on developing new systemic approaches. The patterns of failure following standard treatment for high-risk PCa reveal a large component of local recurrence in addition to distant spread of disease [25,26].

In patients at greater risk for EPE, a BT boost combined with additional EBRT can ensure adequate margin of coverage of surrounding tissues even in the case of minimal spread of disease. As there is mounting evidence that dose escalation leads to a decrease in the rate of treatment failure [27-29], this combined BT and supplemental EBRT is now commonly used to provide a very conformal high-dose-boost to the prostate. Indeed, this synergistic effect of the combination strategy of two types of RT can induce a greater biologically effective dose (BED), the values of which are strongly correlated with treatment outcomes in biochemical control of disease [6,30]. Stone *et al. *have shown that local control improves from 78% to 98% (as determined by biopsy) when the BED is increased from ≤ 150 Gy to > 200 Gy [31]. Our protocol with combination of BT boost and EBRT should deliver similar high doses, thus greatly decreasing the likelihood that local failure will be responsible for future PSA increase. The protocol also allows centers to adjust their EBRT component based on the delivered D90 so the final BED will be between 200 and 220 Gy. This RCT will be the first to test the hypothesis of whether longer use of HT can prevent relapse and death that results from coexistent micrometastases when high-risk PCa is treated with high radiation doses. Zelefsky *et al. *have shown that even 81 Gy of IMRT is associated with a much higher local failure (12%) and that I-125 monotherapy is superior to 81 Gy in both biochemical control and achieving a lower PSA nadir [29].

The combination of HT has an independent cytotoxic effect on prostate cancer cells, and the rationale for combining HT with radiation is to act as a "sensitizer" for radiation to enhance the cytotoxic effect on cancer cells, and to eradicate micrometastatic disease beyond the radiation volume. Several RCTs have documented a prolongation in DSS and/or OS when HT is added to EBRT in comparison to EBRT alone in the treatment of men with localized high-risk and locally advanced PCa [10-12]. Recently, Widmark *et al. *reported that DSS and OS at 10 years were significantly higher with HT plus EBRT than with HT alone [32]. However, as mentioned above these studies were performed with insufficient radiation dose and whether the benefit from the prolonged HT was from a local or distant effect, or a combination of both is unknown. The majority of these prior RCTs were performed with conventional doses of EBRT (65-70 Gy) and it remains to be seen how the studies would turn out if higher IMRT doses were used. However, even 81 Gy of IMRT might prove insufficient to control the local disease in high-risk PCa.

There are several aspects of our study that are similar to the RCTs done with HT for breast cancer. In several trials that directly compared approximately 5 years of tamoxifen with 1 to 2 years involving more than 18,000 women, with a mean length of follow-up of 5 person-years, the estimated risk reductions were 15% (P < 0.001) for recurrence and 8% (P = 0.01) for breast cancer mortality [33]. An important feature for the breast cancer RCTs was the success of the combined modality therapy in treating the primary lesion, lumpectomy and whole breast irradiation. The risk of local recurrence after the primary treatment is less than 5%. Thus the breast cancer RCTs were testing the hypothesis that long term HT would reduce the chance of a breast cancer related death.

Adequate local control has been the challenge when treating locally advanced and high grade PCa. Often patients are offered radical prostatectomy, especially if they are young. However, three recent RCTs involving adjuvant EBRT following RP demonstrated markedly decreased local recurrence and improved survival in the EBRT arm [34-36]. As discussed above, achieving an adequate dose of IMRT to eradicate all local disease is not currently possible. The need for very high local dose was recently shown in a multicenter report in Gleason score 8-10 PCa where patients receiving a BED of > 220 Gy (I-125 implant D90 of 130 Gy combined with 45 Gy EBRT) had a 25% improvement in biochemical control, decreased bone metastases and improved survival when compared to men who received lower dose of irradiation [6].

Relatively few data are available, mostly from single institutions, on the morbidity of this combined modality treatment, despite the increased use of this treatment strategy during the 1990s [37-40]. Since the 1990s, ultrasound-guided transperineal interstitial permanent BT has been a commonly used treatment strategy for patients with PCa [17,41]. In Japan, ^125^I-BT was added to the Japanese armamentarium for treatment of localized PCa in 2003 [15]. Since then, more than 10,000 patients have undergone this procedure. Although there are many benefits of combined use of HT with EBRT, the potential adverse events significantly caused by this treatment should be taken into consideration [13,42]. The PROST-QA study prospectively measured patient-reported QOL outcomes before and after PCa treatment [14]. Sexual function was persistently poorer among radiation patients who received HT than among those who did not. Vitality and other outcomes related to HT (*e.g*., fatigue, weight change, gynecomastia, depression, and hot flashes) were also poorer in the HT patients. In contrast, a meta-analysis of randomized trials suggested that HT plus RT decreases recurrence and mortality rates of patients with high-risk PCa, without affecting toxicity [43].

The optimal duration of concomitant HT for high-risk PCa when combined with dose-escalated RT is not yet known. The period of 3 years of adjuvant HT was chosen empirically; shortening of this period would reduce costs and side effects due to longer HT and may be possible, as patients with locally advanced prostate cancer in the late 1990s had less tumor burden and were younger than those of the mid-1980s. The RTOG study 92-02 demonstrated that the additional 2.5 years of HT to EBRT group showed significant improvement over EBRT alone for all endpoints except OS in comparison to 4 months of HT with EBRT [11]. The EORTC study 22961 also showed that the combination of EBRT plus short-term HT provides inferior survival to EBRT plus long-term HT for 3 years in the treatment of locally advanced PCa [12].

On the other hand, the role of HT in conjunction with BT for high-risk PCa patients is not as clearly defined. Moreover, the ABS also provides no clear indication for adjuvant HT when combination of BT and EBRT are performed for intermediate- to high-risk PCa, except in the aim of downsizing the prostate gland when the initial size surpasses 60 cc [7]. Although some authors have certainly reported clinical advantages to addition of HT to BT [20,44,45], a large retrospective matched-pair analysis failed to show a benefit of HT in conjunction with BT for any risk group, Gleason score, pretreatment PSA level, or clinical stage [46]. Taken together, the lack of evidence from randomized trials has resulted in a variety of treatment approaches performed in both clinical community and academic settings, so that variations in sequence of EBRT and BT, choice of isotope, use of HT, and the experience of reporting institutions have all led to uncertainties in extrapolating reported results. Furthermore, there is still uncertainty regarding not only the adequate duration of HT but also both the optimal timing and contents of HT, with regard to adding HT to EBRT and/or BT in high-risk PCa.

Accordingly, we have designed this TRIP study of trimodality treatment consisting of ^125^I-BT, EBRT, and either short- or long- term HT for high-risk PCa. To our knowledge, there have been no previous reports of prospective studies documenting the efficacy and safety of trimodality therapy for high-risk PCa patients. Initially, we assess the tumor control outcomes of patients treated with this modality (BT and supplemental EBRT with neoadjuvant and concurrent HT) with or without adjuvant HT for 2 years. In this setting, the present multi-institutional TRIP study was designed to determine the efficacy of the combination of EBRT with ^125^I-BT boost in all patients treated with uniform widely accepted guidelines for RT in addition to use of HT. Although Lee *et al. *reported a significant advantage of adding HT to BT in patients with high-risk PCa, improvements in biochemical outcome were restricted to those patients with "low-dose" implants [44]. In addition, the ability to adequately irradiate the periprostatic region using extracapsular seeds may also improve biochemical outcome. Taking into account the potential learning curve for ^125^I-BT, participating institutions in our study were required to have performed at least 50 previous ^125^I-BT procedures, thus mitigating the impact of inexperience with this procedure while being inclusive of the majority of facilities at which ^125^I-BT is performed. In addition a dosimetry assessment committee will review the BT outcomes of all participants. Given the variable nature of delivered implant dose, all centers will be encouraged to irradiate their patients to a dose of 200-220 Gy, in the event the implant dose is too low or high. The uniformity of dose delivery will more closely mimic a much higher, more homogeneous dose of EBRT. Finally, this RCT also provided additional insight regarding the efficacy and limitations of the addition of 2 years of adjuvant HT to this trimodality therapy (dose escalated RT plus HT before and during RT), with evaluation of the primary endpoint of bPFS at 7 years. Insight should be gained as to whether extended HT would benefit the patients who harbor micrometastases when adequate local therapy has been delivered.

## Abbreviations

ABS: American brachytherapy society; bPFS: Biochemical progression-free survival; BED: Biological effective dose; BT: Brachythrerapy; CAB: Combined androgen blockade; CTV: Clinical target volume; 3D-CRT: Three-dimensional conformal radiation therapy; DSS: Disease specific survival; DVH: Dose-volume histogram; ETV: Evaluation target volume; EBRT: External beam radiation therapy; EPE: Extraprostatic extension; EPIC: Expanded prostate cancer index composite; IMRT: Intensity modulated radiation therapy; IPSS: International prostate symptom score; OS: Overall survival; LH-RHa: Luteinizing hormone-releasing hormone-agonist; RCT: Randomized controlled trial; PCa: Prostate cancer; PTV: Planning target volume; TRI: Translational research informatics center; TRUS: Transrectal ultrasound; QOL: Quality of life.

## Competing interests

The authors declare that they have no competing interests.

## Authors' contributions

HK, SS, AY, SE, MF, TD, HY, and MN planned, coordinated and conducted the study. Medical care was provided by HK, SS, AY, SE. and MN, KM provided randomization. HT contributed pathological diagnosis. MF, TD, HY, and NS took part in conducting the study.

The scientific program was planned by HK, SS, AY, NS, SE, MF and MN, and carried out by MN. All authors read and approved the final manuscript. All other participants in this study contribute to the enrollment, treatment and follow up of patients.

## Pre-publication history

The pre-publication history for this paper can be accessed here:

http://www.biomedcentral.com/1471-2407/12/110/prepub
